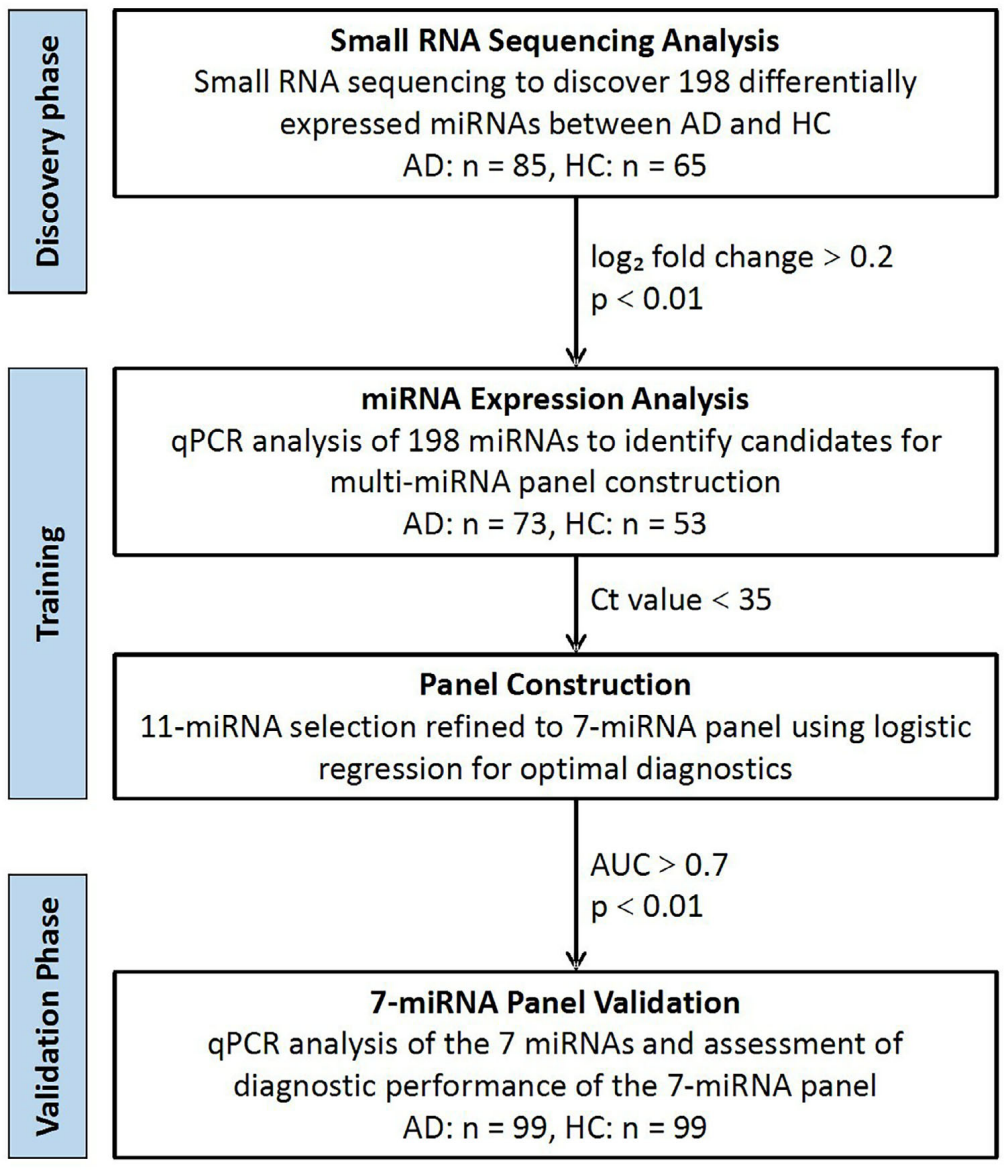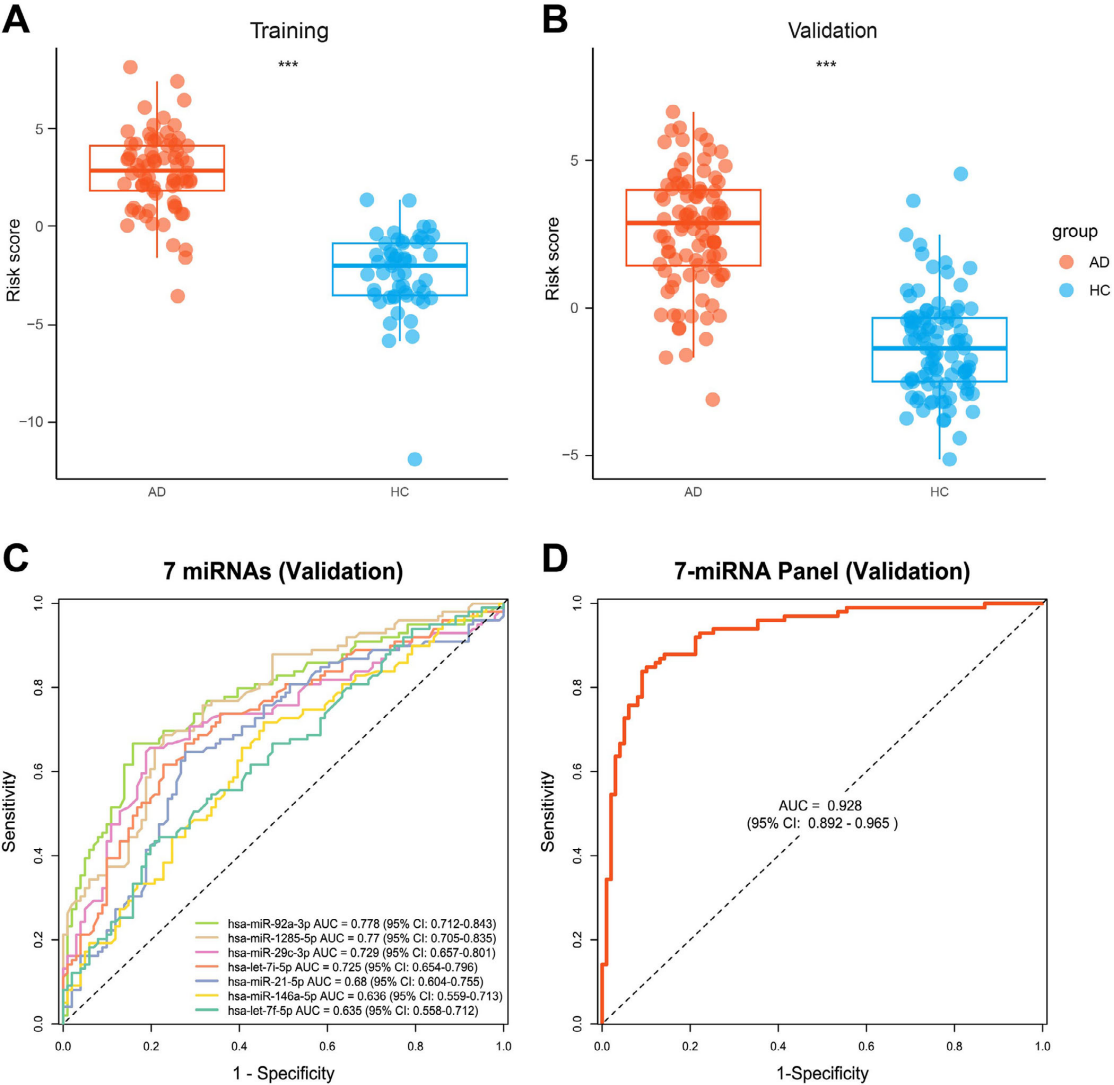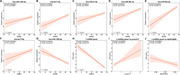# Development of a Serum‐based miRNA Panel for Alzheimer's Disease Diagnosis

**DOI:** 10.1002/alz70856_100210

**Published:** 2025-12-25

**Authors:** Xinyi Xia, Xinyu Zhang, Qi Qin, Chang Su, Yi Tang

**Affiliations:** ^1^ Department of Neurology & Innovation Center for Neurological Disorders, Xuanwu Hospital, Capital Medical University, National Center for Neurological Disorders, Beijing, Beijing, China; ^2^ Miracle (Shenzhen) Biotechnology Co., Ltd., Shenzhen, Guangdong, China; ^3^ Neurodegenerative Laboratory of Ministry of Education of the People's Republic of China, Beijing, Beijing, China

## Abstract

**Background:**

MicroRNAs (miRNAs) are emerging as promising blood‐based biomarkers for Alzheimer's disease (AD) due to their stability and regulatory roles in disease‐related pathways. This study aimed to develop a serum‐based miRNA panel for AD diagnosis.

**Method:**

Serum samples from 474 participants were categorized into discovery (85 AD, 65 healthy controls [HC]), training (73 AD, 53 HC), and validation (99 AD, 99 HC) cohorts. We identified a 7‐miRNA panel using small RNA sequencing and qPCR, validated with machine learning.

**Result:**

The 7‐miRNA panel achieved area under the curve values of 0.970 and 0.928 in the training and validation cohort respectively. The risk score derived from the 7‐miRNA panel was significantly associated with cognitive impairment (MMSE scores, r = ‐0.72) and plasma amyloid pathology biomarkers (Aβ42/40 ratio, r = ‐0.25; *p*‐tau217, r = 0.36).

**Conclusion:**

The serum‐based 7‐miRNA panel demonstrates strong diagnostic potential for AD, offering a minimally invasive and accessible approach.